# Digit ratios and their asymmetries as risk factors of developmental instability and hospitalization for COVID-19

**DOI:** 10.1038/s41598-022-08646-7

**Published:** 2022-03-17

**Authors:** A. Kasielska-Trojan, J. T. Manning, M. Jabłkowski, J. Białkowska-Warzecha, A. L. Hirschberg, B. Antoszewski

**Affiliations:** 1 Plastic, Reconstructive and Aesthetic Surgery Clinic, Institute of Surgery, Medical University of Lodz, Kopcinskiego 22, 90-153, Lodz, Poland; 2 Applied Sports, Technology, Exercise, and Medicine (A-STEM), Swansea University, Swansea, UK; 3 Department of Infectious and Liver Diseases, Medical University of Lodz, Lodz, Poland; 4 Department of Gynecology and Reproductive Medicine, Karolinska University Hospital, Stockholm, Sweden

**Keywords:** Biomarkers, Diseases

## Abstract

COVID-19 presents with mild symptoms in the majority of patients but in a minority it progresses to acute illness and hospitalization. Here we consider whether markers for prenatal sex hormones and postnatal stressors on developmental instability, i.e. digit ratios and their directional and unsigned asymmetries, are predictive of hospitalization. We focus on six ratios: 2D:3D; 2D:4D; 2D:5D; 3D:4D; 3D:5D; 4D:5D and compare hospitalized patient and control means for right, and left ratios, directional asymmetries (right–left) and unsigned asymmetries [|(right–left)|]. There were 54 patients and 100 controls. We found (i) patients differed in their digit ratios from controls (patients > controls) in all three ratios that included 5D (2D:5D, 3D:5D and 4D:5D) with small to medium effect sizes (*d* = 0.3 to 0.64), (ii) they did not differ in their directional asymmetries, and (iii) patients had greater |(right–left)| asymmetry than controls for 2D:4D (*d* = .74) , and all ratios that included 5D; 2D:5D (*d* = 0.66), 3D:5D (*d* = .79), 4D:5D (*d* = 0.47). The Composite Asymmetry of the two largest effects (2D:4D + 3D:5D) gave a patient and control difference with effect size *d* = 1.04. All patient versus control differences were independent of sex. We conclude that digit ratio patterns differ between patients and controls and this was most evident in ratios that included 5D. Large |(right–left)| asymmetries in the patients are likely to be a marker for postnatal stressors resulting in developmental perturbations and for potential severity of COVID-19.

## Introduction

Severe acute respiratory syndrome coronavirus 2 (SARS-CoV-2) causes a respiratory and systemic illness (COVID-19) which may present as a severe pneumonia in 10–15% of patients. Severe disease can lead to acute respiratory distress and multi-organ failure often followed by intravascular coagulopathy^1,2^. Due to this variety and unknown severity and death risk factors, many studies and analyses have focused on identifying biomarkers of severe disease or poor outcomes in COVID-19 infections. Recent studies have shown that the clinical progress could be severe in cases of increased: neutrophil-lymphocytes ratio, C-reactive protein (CRP), troponin I, lactate dehydrogenase and that the troponin I, elder age and SO_2_ values are linked to in-hospital mortality. Across nations, there is variation in case fatality rates and in predictors of mortality^3^. For example, data from Belgium indicated severity was associated with older age, renal insufficiency, higher lactate dehydrogenase and thrombocytopenia and obesity^4^. Patterns of severity from Chinese studies included higher age, male sex, higher Body Mass Index, hypertension, lower T lymphocyte and B lymphocyte count, higher white blood cell count, higher D2 dimer, procalcitonin, CRP and aspartate aminotransferase. Among these variables age and weight appeared to be independent risk factors for disease severity^5^. Importantly, identifying these risk factors did not significantly change our understanding of the COVID-19 pandemic nor did it facilitate a reduction in mortality.

In many populations the severity of COVID-19 is sex dependent (males > females)^6^. The excess of male deaths has led to two opposing suggestions: (i) The androgen-driven COVID-19 pandemic theory^7,8^, and (ii) the male hypogonadism theory^9^. With regard to support for the former, viral entry to cells is androgen dependent, involving priming of the spike proteins and cleaving of angiotensin converting enzyme 2 (ACE2). Both processes are facilitated by trans-membrane protease, serine 2 (TMPRSS2)^10^. Androgen receptor activity is a requirement for the transcription of the TMPRSS2 gene, suggesting that testosterone facilitates SARS-CoV2 cell entry^11^. Thus the androgen-driven COVID-19 pandemic theory postulates that high mortality from SARS-CoV2 in men is related to hypergonadism. In contrast, proponents of the male hypogonadism theory point to theory-inconsistent relationships between testosterone and COVID-19 in males^9^. Thus, in men COVID-19 mortality rates increase with age but testosterone levels decrease^12^. The male hypogonadism theory gave a rationale for the analyses conducted by Manning and Fink^9^ who considered national values of digit ratios, in this case 2^nd^ to 4^th^ digit ratio, in relation to Covid-19 case fatality rates (CFR’s). The relative lengths of the second digit and fourth digit (digit ratio or 2D:4D) is sexually dimorphic (2D:4D males < 2D:4D females). It has been suggested that 2D:4D is a biomarker of prenatal sex steroids exposure, i.e. low 2D:4D correlates with high prenatal testosterone and low oestrogens, while high 2D:4D correlates with low foetal testosterone and high oestrogen. There is considerable support for the link between 2D:4D and prenatal sex steroids^13^ but for a contrasting view see McCormick & Carre, 2020^14^. Manning and Fink found that nations with high CFR’s had high mean male 2D:4D^9^, thus supporting the hypogonadal theory (in support see Sahin, 2020 and for a critical view see Jones et al., 2020^15,16^). With regard to right–left asymmetry of 2D:4D, i.e. directional asymmetry of 2D:4D, (Δ r–l 2D:4D) is also thought to be a negative correlate of high prenatal testosterone and low prenatal oestrogen^9,17–21^. In general unsigned asymmetries (such as that of single digit R–L asymmetries) may reflect developmental instability related to postnatal stressors including sex steroids and to correlates of low socio-economic status such as poor nutrition^22,23^.

Sex differences in digit ratios, with males < females, are present across a number of digits^24–27^. Here we focus on six ratios from digits 2 to 5, i.e. 2D:3D, 2D:4D, 2D:5D, 3D:4D, 3D:5D and 4D:5D (digit 1 is difficult to measure accurately). There is considerable evidence that prenatal sex steroids have an effect on 2D:4D. However, effect sizes for 2D:4D are likely to be linked to other ratios that share 2D or 4D. Right 2D:4D is stable during growth in children and adolescents supporting the contention that it retains information pertaining to prenatal sex steroids. However, other ratios, such as those that include 5D (and in particular 3D:5D), show sex differences (males < females) but are highly unstable during growth in children and adolescents. This instability is present in both hands but is expressed most intensely in the left hand^24,27^. The difference in stability across right and left hands suggests that R–L differences in ratios may contain important information which pertains to developmental instability rather than effects of prenatal sex steroids. Therefore, for each ratio we consider values from the right hand, left hand, Δ right–left (directional asymmetry) and |(right–left)|(FA). The purpose of this preliminary report was to focus on associations between digit ratios and severity of COVID-19, as evidenced by hospitalization of patients.

Following from the across-nation correlations between digit ratio and CFR’s we suggest that, in comparison to controls, patients hospitalized for COVID-19 will have: (i) high right and left hand digit ratios, and high Δ right–left directional asymmetry, indicating exposure to low prenatal testosterone and high prenatal oestrogen, and (ii) high |(right–left)| unsigned asymmetry (FA), indicating heightened levels of developmental instability arising from stressors such as pubertal sex steroids. With regard to these predictions we emphasize that there is potential for considerable inter-correlation between digit ratios. In this regard, 2D:4D has been shown to exhibit developmental stability while 3D:5D is particularly unstable during development^24,27^. Therefore, the patterns associated with 2D:4D and 3D:5D are least likely to be affected by inter-correlations between digit ratios. Thus, 2D:4D may contain information concerning prenatal influences and 3D:5D information concerning postnatal effects of developmental instability. Consequently, we suggest these two digit ratios should be the focus of greatest attention.

## Methods

Participants were recruited from a Department of Infectious Diseases and Liver Diseases of a Medical University. All consecutive patients with diagnosed COVID-19 who were hospitalized in the Department due to the severe or high risk of severe COVID-19 were included. During a first wave of the Covid-19 pandemic (March–August 2020) there were 54 (28 men and 26 women) patients who met the study criteria (Inclusion Criteria: 1. admitted to hospital because of Covid-19, 2. positive PCR test, 3. conscious and able to give informed written consent for participation; Exclusion Criteria: 1. unconscious, unable to give written consent for participation, 2. Covid-19 positive patients hospitalized because of other than Covid health issues, pregnant women, children (< 18 years), patients after transplantations, during immunotherapy and with renal failure requiring dialyses). Of these, there were 51 for whom the right hand ratios could be measured, 52 for the left hand and 49 for whom R–L measurements were possible (one hand only was available for measurement for 5 patients due to a hand injury and/or finger contractures). The protocol of the study included a clinical questionnaire based on medical records (age, symptoms) severity of the disease (scale 0–4; 0 -no symptoms, 1—mild, 2—medium, 3—severe, 4—critical), length of hospitalization and oxygen therapy, days in intensive care unit, concomitant diseases, history of smoking and occupational exposure, and laboratory test results (white blood count, fibrinogen, d-dimers, platelets count, oxygen saturation, procalcitonin) and anthropometric measurements.

Controls, 47 women (mean age 51.3 ± 16.1 years) and 53 men (mean age 52.2 ± 14.4 years) were recruited from a Plastic Surgery Out-patient Clinic (approximately 80% of the sample) and among other volunteers after the first wave of COVID-19. We consider our sample to be representative of the general population. Thus, the Out-patient Clinic is state-funded, attendees are from a variety of backgrounds and ages, and they present with a variety of needs such as removal of scars, moles and eyelid disorders (ptosis, ectropion, entropion). We did not include women after breast cancer who come for breast reconstruction, patients with skin cancer, post-bariatric patients, any patient who has immunosuppression. Controls were included based on a negative history of COVID-19 (non-infected or non-symptomatic subjects). One woman reported injury of the 3^rd^ finger of the left hand and was included in the study after exclusion of this finger measurement. All the participants were White (based on patients’ medical data and controls recruitment).

### Ethical statement

The protocol was agreed by the Bioethical Committee of the Medical University of Lodz (RNN/152/20/KE). All methods were performed in accordance with the relevant guidelines and regulations. Written informed consent was obtained from all participants.

### Hand images

With regard to the measurement of digit ratio, our preference would have been for direct measurement of fingers. However, it was difficult to measure digit length directly from the hands of the patients because many of them were very ill and measurers were hampered by personal protective equipment. Moreover, direct digit measurement requires a period of time during which the patient and measurer are in close proximity. This is to be avoided with an infectious viral agent. Indirect methods such as the use of photocopies or scanners, give a permanent record of digit lengths. Against this, it was felt that the use of photocopiers or scanners was not appropriate as repeated use of such machines may result in cross infection resulting from virus particles being left on surfaces. Moreover, in comparison to directly measured digits, indirect images yield lower 2D:4D ratios^24,28,29^ with magnitudes that may vary by sex and hand^30^. These effects may extend to asymmetries also, and the accuracy of asymmetries measured from photocopies has been questioned^31^. Therefore, it was decided to photograph the hands of the patients. Typically, the patient was sitting up in bed and he/she was instructed to place their hands horizontally with the palms uppermost, the digits straight and together. In order to minimize inconvenience to the patient it was decided not to use a tripod with the camera. Rather, the experimenter held the camera approximately 30 cm above the patient’s hand. This protocol was felt to be appropriate as it would minimise the amount of proximity necessary between experimenter and patient. Moreover, it gives a permanent image of the supine hand which did not involve potential distortions resulting from digit contact with glass surfaces. It is to be noted that the relative lengths of digits within a hand can be obtained in this way but R–L contrasts of absolute measures of digit length are likely to be unreliable as they will be influenced by small vertical differences in distance between hand and camera. Photographs were checked for definition at the tips of the digits and at the metacarpophalangeal crease at the base of the digits. A second photograph was taken if the first was not deemed to be of sufficient quality.

### Measurements

Eight measurements were taken from patients’ and controls’ hand photographs: second, third, fourth and fifth digits’ lengths (2D, 3D, 4D and 5D) (right (R) and left hand (L)). On the basis of the these parameters the following ratios were calculated: 2D:3D, 2D:4D, 2D:5D, 3D:4D, 3D:5D, 4D:5D for the right (R) and left (L) hand (D length [mm]/D length [mm]) in addition to the ratios’ directional asymmetries (right ratio–left ratio: Δ2D:3D, Δ2D:4D, Δ2D:5D, Δ3D:4D, Δ3D:5D, Δ4D:5D) and unsigned asymmetries (FAs) (|(right–left)|). We also calculated two composite asymmetries by summing (i) all six (|(right–left)|) asymmetries and (ii) and asymmetries for the “independent” ratios of 2D:4D and 3D:5D. We refer to the latter as the “Clinical Composite Asymmetry” in the Results section. All measurements (in patients and controls) were made twice by AKT using the GNU Image Manipulation Program (GIMP) version 2.10.20. For a subset of measurements, a sliding calliper was used directly on the image of the fingers on the computer screen (by JTM). Measurements were performed on the palmar side of the hand using anthropometric points lying on the digit axis: pseudophalangion—the most proximal point in the finger metacarpophalangeal crease, dactylion—the most distal point on the fingertip^32^. There was high repeatability of digit ratios within and between observers. The final ratios were calculated as a mean of two ratios obtained from the GIMP program. These ratios were used in the further analysis of the data.

### Statistical analysis

Analysis was conducted on the differences in the digit ratios and their directional (right–left) and unsigned [|(right–left)|] asymmetries between patients hospitalized due to Covid-19 and controls. The normality of distribution of the tested variables was examined (using Shapiro–Wilk test) and the homogeneity of variances was checked (using the Bartlett test). With both assumptions met we applied univariate t-tests for differences between means in addition to two-way analysis of variance (ANOVA). If any of these assumptions were not met then non-parametric tests were used. Logistic regression was used to evaluate the relationship between the asymmetry index being the sum of the unsigned asymmetries of the ratios of the largest effect sizes estimated with omega-squared for ANOVA (“Clinical Composite Asymmetry”) and the risk of hospitalization due to Covid-19. Finally, logistic regression model included the following variables: the sum of asymmetries of 2D:4D and 3D:5D (dependent variable) and the group (patient vs. control) and sex (independent variables). Effect size for inter-group differences was evaluated with Cohen’s *d* for *t*-tests and omega-squared (ω^2^) for ANOVA. The interpretation of descriptors of magnitude for the former were small 0.20, medium 0.50 and large 0.80 and for ω^2^ > 0.01 —weak, > 0.06—medium, > 0.14—strong effect. The probability of *p* < 0.05 was accepted as a level of significance.

## Results

### Characteristics of Covid-19 patients

Among 54 patients there were 28 men (mean age 54.7 ± 14.7 years) and 26 women (mean age 59.3 ± 18.2 years). The group of patients did not differ in age and frequency of males and females from the controls (*F* = 1.085; *p* = 0.299). Specific characteristics (i.e. BMI, comorbidity, smoking status) and Covid-19 symptoms and severity are shown in Table 1.

**Table 1:** Characteristics of patients hospitalized because of Covid-19.

n (%)	Men (n = 28)	Women (n = 26)	All (n = 54)
Age [years]	54.7 ± 14.7	59.3 ± 18.2	56.9 ± 16.5
BMI [kg/m^2^]	30.3	30.4	30.3
**Covid-19 symptoms**			
Dyspnoea	16 (57.1)	18 (69.2)	34 (63)
Cough	19 (67.8)	20 (76.9)	39 (72.2)
Fever	21 (75)	24 (92.3)	45 (83.3)
Fatigue	18 (64.3)	22 (84.6)	40 (74.1)
Loss of smell and taste	1 (3.6)	3 (11.5)	4 (7.4)
Other/subclinical	4 (14.3)	–	4 (7.4)
Severity [mean] ( Likert scale 1–4)	2.2	1.64	1.9
Length of: hospital stay/oxygen therapy [mean, days]	15.8/8.2	14.6/5.1	14.9/7.8
Hypertension	9 (32.1)	16 (61.5)	25 (46.3)
Ischaemic heart disease	7 (25)	9 (34.6)	16 (29.6)
Diabetes mellitus	3 (10.7)	5 (19.2)	8 (14.8)
Lung disorder (asthma, COPD)	2 (7.1)	2 (7.7)	4 (7.4)
Thyroid disorders	2 (7.1)	1 (3.8)	3 (5.6)
Other (cancer, hematologic, autoimmune disease)	3 (10.7)	3 (11.5)	6 (11.1)
Smoking/ex-smoking	8 (28.6%)/12 (42.8%)	2 (7.7%)/6 (23.1%)	10 (17.9)/18 (33.3)

### Reliability of measurements

First we checked intra-observer reliability for all twelve ratios (ratio 1 versus ratio 2) for observer AKT. The coefficient of reliability for raw measurements (R) ranged from 96.07% (for 3D:4D L) to 99.66% (for 2D:5D R). Intra-class correlation coefficients were also very high Table 2). Repeatability of signed asymmetries can be low because they contain the measurement error of four digits. However, for the signed asymmetries (R–L) and the unsigned asymmetries (|R–L|) the R ranged from 99.86% (for 2D:3D |R–L| to 99.97% (for 2D:4D R–L) also with high ICC’s (Table 2). Further analysis included mean values of ratio 1 and 2. Then, inter-observer reliability was checked (observer AKT versus observer JTM), for two ratios: 2D:4D R and 2D:4D L and their signed and unsigned asymmetries. Due to the high reliability between observers (2D:4D R: TEM = 0.0089, R = 99.66%, ICC = 98.09%; 2D:4D L: TEM = 0.0118, R = 98.91%, ICC = 97.93%; R–L: TEM = 0.0076, R = 98.66%, |R–L|: TEM = 0.0076, R = 96.13%, ICC = 96.43%) final analysis included data from AKT.

**Table 2:** Technical error measurement (TEM) and the coefficient of reliability for raw measurements (R) for ratios and for R–L and |R–L| of six ratios for observer 1.

Intra-observer Reliability	TEM	R (%)	ICC (%)
2D3D R 1 vs. 2	0.005	97.89	97.91
2D3D L 1 vs. 2	0.009	95.61	95.65
2D4D R 1 vs. 2	0.004	99.41	99.42
2D4D L 1 vs. 2	0.004	99.63	99.63
2D5D R 1 vs. 2	0.009	99.66	99.66
2D5D L 1 vs. 2	0.012	98.91	98.92
3D4D R 1 vs. 2	0.006	98.18	98.19
3D4D L 1 vs. 2	0.009	96.07	96.11
3D5D R 1 vs. 2	0.011	99.52	99.52
3D5D L 1 vs. 2	0.0110	98.77	98.78
4D5D R 1 vs. 2	0.008	99.43	99.44
4D5D L 1 vs. 2	0.011	97.43	97.46
2D3D R1–L1 vs. R2–L2	0.0100	99.88	99.88
2D3D |R1–L1| vs. |R2–L2|	0.0096	99.86	99.87
2D4D R1–L1 vs. R2–L2	0.0053	99.97	99.97
2D4D |R1–L1| vs. |R2–L2|	0.0052	99.96	99.96
2D5D R1–L1 vs. R2–L2	0.0138	99.89	99.89
2D5D |R1–L1| vs. |R2–L2|	0.0122	99.88	99.88
3D4D R1–L1 vs. R2–L2	0.0111	99.89	99.89
3D4D |R1–L1| vs. |R2–L2|	0.0091	99.91	99.91
3D5D R1–L1 vs. R2–L2	0.0148	99.89	99.89
3D5D |R1–L1| vs. |R2–L2|	0.0143	99.87	99.87
4D5D R1–L1 vs. R2–L2	0.0126	99.90	99.90
4D5D |R1–L1| vs. |R2–L2|	0.0120	99.89	99.89

### Digit ratios: patients vs. controls

There were no relationships between age and digit ratios in any of the twelve tests (values of *r* varied from − 0.14 for right 2D:3D to 0.1 for right 4D:5D, all p > 0.05).

Patient and control means and SD’s for six ratios and 12 effects (right and left ratios) are given in Table 3. Values of *p* and Cohen’s *d* are included from *t*-tests. There were five significant effects ranging from small to medium in magnitude. Four of these showed higher values in the patients compared to the controls, i.e. 3D:5D right *d* = 0.55, left *d* = 0.37; 4D:5D right *d* = 0.64, left *d* = 0.58. One effect showed mean patient < control (right 2D:5D *d* = 0.38). We note that all five significant effects were present in ratios that included 5D. Correction for multiple tests is inappropriate across Table 3 as the variables are not independent, i.e. the length of each digit is present in three ratios. We considered the effect of sex on these patient/control differences by performing two-factor ANOVA’s (independent variables: group [patients, controls], sex [males, females] with dependent variable digit ratio). All five remained significant (see effect sizes [ω^2^]), There were no effects of sex and no significant interactions (Table 4).

**Table 3:** Patient and control means and SD’s for six digit ratios (2D:3D; 2D:4D; 2D:5D;3D:4D; 3D:5D; 4D:5D) and their signed and unsigned asymmetries.

	Patients			Controls			*p*	*Cohen’s d*
	*n*	mean	SD	*n*	mean	SD		
**2D:3D**								
Right	51	0.89	0.036	100	0.900	0.035	0.114	− 0.27
Left	52	0.897	0.044	99	0.897	0.038	0.987	0
R–L	49	− 0.003	0.041	99	0.003	0.037	0.403	− 0.14
|R–L|*	49	0.033	0.024	99	0.027	0.025	0.164	0.21
**2D:4D**								
Right	51	0.962	0.054	100	0.969	0.046	0.386	− 0.15
Left	52	0.968	0.067	100	0.974	0.042	0.583	− 0.1
R–L	49	− 0.005	0.065	100	− 0.005	0.039	0.95	− 0.01
|R–L|*	49	0.053	0.037	100	0.029	0.027	** < 0.0001**	**0.74**
**2D:5D**								
Right	51	1.259	0.152	100	1.213	0.079	**0.047**	**0.38**
Left	52	1.232	0.113	100	1.202	0.078	0.103	**0.3**
R–L	49	0.031	0.13	99	0.011	0.066	0.314	0.19
|R–L|*	49	0.096	0.092	100	0.049	0.045	**0.004**	**0.66**
**3D:4D**								
Right	51	1.081	0.045	100	1.078	0.036	0.63	0.08
Left	52	1.079	0.047	99	1.087	0.040	0.314	− 0.17
R–L	49	− 0.002	0.051	99	0.001	0.115	0.395	0.15
|R–L|*	49	0.037	0.034	99	0.031	0.032	0.557	0.16
**3D:5D**								
Right	51	1.414	0.154	100	1.348	0.069	**0.005**	**0.55**
Left	52	1.372	0.099	99	1.342	0.066	**0.047**	**0.37**
R–L	49	0.039	0.119	99	0.008	0.051	0.079	**0.35**
|R–L|*	49	0.091	0.085	99	0.039	0.034	** < 0.0001**	**0.79**
**4D:5D**								
Right	51	1.306	0.107	100	1.252	0.056	**0.001**	**0.64**
Left	52	1.271	0.067	100	1.235	0.059	**0.001**	**0.58**
R–L	49	0.037	0.092	100	0.017	0.050	0.168	**0.26**
|R–L|*	49	0.066	0.074	100	0.038	0.036	**0.013**	**0.47**
**Composite Asymmetry**	49	0.375	0.248	99	0.213	0.142	< 0.0001	**0.8**
**Clinical Composite Asymmetry** |Δ2D:4D| + |Δ3D:5D|	49	0.143	0.092	99	0.068	0.046	< 0.0001	**1.04**

Values of *p* and Cohen’s *d* are given for differences between patients and controls (t test). Negative *d* denotes lower values in patients compared to controls and positive *d* denotes higher values in patients compared to controls. All significant patient/control differences (in bold) involve digits 2D and 5D and all have positive values of d, i.e. patients > controls.

*Mann–Whitney test.

**Table 4:** Differences in digit ratios and their asymmetries between patients and controls—(ANOVA) controlled for sex.

	Group		Sex		Group*sex (interaction)		ES** (ω^2^)
	F	p	F	p	F	p	
**2D:3D** R	2.604	0.1087	1.354	0.246	0.683	0.410	
Left	0.000	0.9845	1.224	0.270	1.421	0.235	
R–L	0.625	0.4306	0.569	0.452	1.253	0.265	
|R–L|	1.580	0.2107	0.352	0.554	1.659	0.200	
**2D:4D** R	0.741	0.3908	0.008	0.930	0.086	0.770	
Left	0.402	0.5272	1.210	0.273	1.223	0.270	
R–L	0.000	1.0000	1.562	0.213	1.106	0.295	
|R–L|	20.146	** < 0.0001**	0.005	0.942	0.106	0.745	**0.115**
|R–L|*	Z = 4.123; p < **0.0001**						
**2D:5D** R	5.809	**0.0172**	0.223	0.638	0.002	0.963	0.031
Right *	Z = 1.639; p = 0.1013						
Left	3.331	0.0700	2.303	0.131	0.550	0.459	
R–L	1.751	0.1878	1.436	0.233	0.883	0.349	
|R–L|	18.281	** < 0.0001**	0.023	0.880	0.001	0.973	**0.105**
|R–L|*	Z = 2.855; p = **0.0043**						
**3D:4D** R	0.264	0.6080	1.140	0.287	0.225	0.636	
Left	1.002	0.3186	0.019	0.891	0.031	0.861	
R–L	0.780	0.3785	0.333	0.565	0.034	0.854	
|R–L|	1.071	0.3024	4.133	**0.044**	1.299	0.256	
**3D:5D** R	12.908	**0.0004**	0.000	0.997	0.108	0.744	**0.074**
Right*	Z = 3.012; p = **0.0026**						
Left	5.010	**0.0267**	1.308	0.255	0.000	0.994	0.029
Left*	Z = 1.189; p = 0.2346						
R–L	5.334	**0.0223**	0.811	0.369	0.101	0.751	0.026
R–L*	Z = 1.964; p = **0.0495**						
|R–L|	27.966	** < 0.0001**	1.664	0.199	0.501	0.480	**0.155**
|R–L|*	Z = 4.841; p** < 0.0001**						
**4D:5D** R	16.564	**0.0001**	0.555	0.457	0.001	0.970	**0.094**
Right*	Z = 3.409; p = 0.0007						
Left	11.712	**0.0008**	1.161	0.283	0.001	0.978	
R–L	2.850	0.0935	0.086	0.770	0.009	0.924	
|R–L|	9.575	**0.0024**	1.071	0.302	0.120	0.730	0.055
|R–L|*	Z = 2.475; p = **0.0133**						
**CA**	25.856	** < 0.0001**	1.374	0.243	0.086	0.769	**0.145**
CA*	Z = 4.494; p < **0.0001**						
**Clinical CA**	44.888	< **0.0001**	1.162	0.283	0.217	0.642	**0.231**
Clinical CA*	Z = 5.676; p < **0.0001**						

*non-parametric test (Mann–Whitney), ** effect size—ω^2^ , CA—Composite Asymmetry |Δ2D:3D| + |Δ2D:4D | + |Δ2D:5D| + |Δ3D:4D | + |Δ3D:5D| + |Δ4D:5D |, Clinical CA –|Δ2D:4D| + |Δ3D:5D| , bold—significant patient/control differences, medium/strong ES.

### Digit ratio asymmetries: patients vs. controls

Two associations between age and asymmetry were significant (|R–L| 2D:4D, *R* = 0.17, *p* = 0.03 and |R–L| 4D:5D, *R* = 0.24, *p* = 0.03). However, there were no relationships between age and asymmetries in ten of the twelve tests (values of *R* varied from − 0.13 for R–L 2D:3D to 0.16 for |R–L| 2D:3D, all p > 0.05).

There were no significant differences in directional asymmetries (R–L) between patients and controls (Tables 3 and 4). There is some evidence in the literature that directional asymmetry of 2D:4D shows sex differences (males < females). Therefore we checked for directional asymmetry (deviations from a mean of zero) in (R–L) in patients and controls for all six ratios split by sex. For male patients (n = 23) one-sample t-tests with mean set at zero showed there were no significant deviations from zero in any ratio (means varied from 0.002 for 3D:4D to 0.049 for 2D:5D, all p > 0.05). For female patients (n = 26), for five ratios means varied from − 0.014 for 2D:4D to 0.031 for 3D:5D, all p > 0.05. For female 4D:5D there was directional asymmetry with mean of 0.034, t = 2.20, p = 0.04. With regard to controls (males n = 53, females n = 47) there was a similar pattern with evidence of directional asymmetry in 4D:5D (males: mean = 0.018, t = 2.44, p = 0.02 and females: mean = 0.016, t = 2.31, p = 0.03). For the remaining ratios means varied from − 0.006 to 0.010, all p > 0.05. Therefore, there was no evidence of significant directional asymmetry in male and female mean (R–L) ratios with the exception of 4D:5D which showed some evidence of higher ratios in the right hand compared to left hand. This suggests that the ratios we consider here (with the exception of 4D:5D) have a mean that does not significantly deviate from zero, i.e. they have the properties of ideal fluctuating asymmetry.

**Figure 1:**
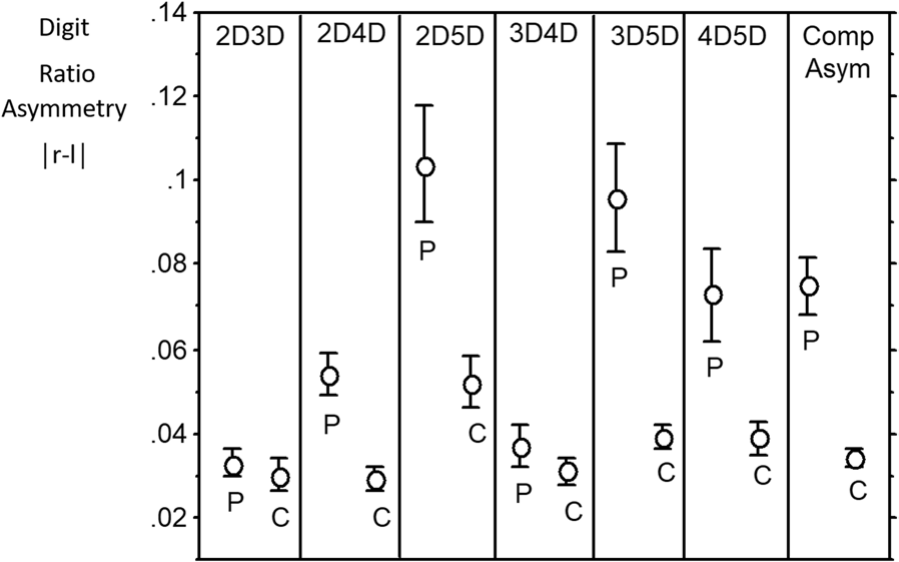
Means (SE error bars) patient (P) and control (C) unsigned asymmetries (|right–left)|) for the digit ratios of 2D:3D; 2D:4D; 2D:5D; 3D:4D; 3D:5D and 4D:5D. Clinical composite asymmetry (Comp Asym) is (|right–left)|2D:4D + |right–left)| 3D:5D)/2.

With regard to unsigned asymmetries (|R–L|), the distributions are “half-normal”. It may be that t-tests of means for patients versus controls are robust enough to give meaningful *p* values. However, in order to consider such differences in a conservative manner we applied Mann–Whitney U tests. There were four significant effects (2D:4D, *d* = 0.74; 2D:5D, *d* = 0.66; 3D5D, *d* = 0.79; 4D:5D, *d* = 0.47) and all showed patients > controls. We note that three effects are for variables that include digit 5D. Summing the unsigned asymmetries across all six ratios we found this composite measure of asymmetry was higher in the patients compared to controls (*d* = 0.8). We then focused on |R–L| in the two “independent” ratios with the highest effect size (i.e. 2D:4D and 3D:5D) and found they were not correlated (*r* = − 0.047). A composite of these two variables, a “Clinical Composite Asymmetry showed the highest effect size of all with patients > controls, *d* = 1.04 (Fig. 1, Table 3).

**Figure 2:**
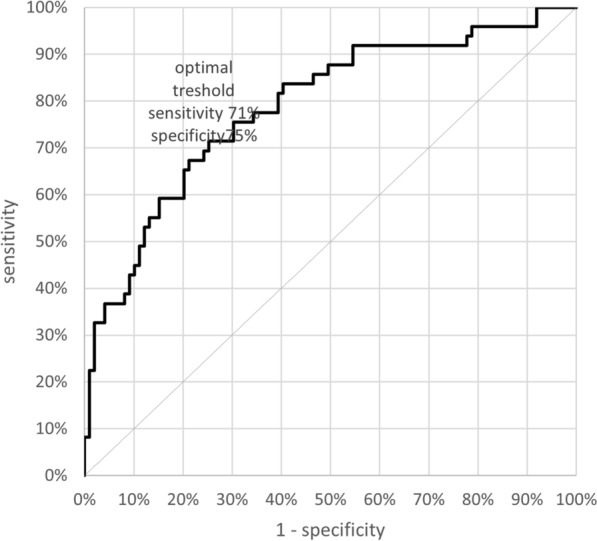
ROC curve for the clinical composite asymmetry predicting probability of hositalization due to Covid-19.

We further considered the effect of sex on these patient/control differences by performing two-factor ANOVA’s (independent variables: group [patients, controls], sex [males, females] with dependent variable digit ratio). There were high effect sizes (ω^2^) for |Δ2D:4D| = 0.115; |Δ2D:5D| = 0.105; |Δ3D:5D| = 0.155; |Δ4D:5D| = 0.055. The effect size for the “Clinical Composite Asymmetry” of 2D:4D and 3D:5D was 0.231. Logistic regression indicated that the “Clinical Composite Asymmetry”, regardless of sex, correlates with the risk of hospitalization due to Covid-19. The area under an ROC curve (AUC) is 0.787, which shows that this classifier is better than a random classifier (AUC = 0.5) with the cut-off point of 0.087. A “Clinical Composite Asymmetry” that is higher than 0.087 discriminates hospitalized patients (sensitivity—71% and specificity 75%) (Fig. 2). The risk of hospitalization in case of the index > 0.087 is 3.5 times higher than in those with lower “Clinical Composite Asymmetry” (OR 3.667).

“Clinical Composite Asymmetry” did not correlate with Covid-19 severity (R = − 0.075, p = 0.61) or with length of hospitalization (R = 0.137, p = 0.35).

## Discussion

This study focused on associations between digit ratios and severity of COVID-19, as evidenced by hospitalization of patients. Our results indicate that digit ratios, and their asymmetries may be regarded as simple clinical markers of the possible risk of hospitalization due to Covid-19. Additionally, the study aimed to examine the role of prenatal sex steroids and that of postnatal developmental instability on the course of Covid-19. We have found evidence for digit ratio and digit ratio asymmetry differences between hospitalized patients with COVID-19 and controls. For digit ratios the magnitude of the effect sizes was small to medium (*d* = 0.3–0.64) and involved all ratios that included 5D, i.e. 2D:5D, 3D:5D and 4D:5D (patients > controls). There were no significant differences between patients and controls for directional (right-left) asymmetry. The largest effect sizes (medium to large) were found for measures of developmental instability, i.e. differences in unsigned asymmetries between patients and controls (patients > controls). These included 2D:4D (*d* = 0.74), and all ratios that involved 5D, i.e. 2D:5D (*d* = 0.66), 3D:5D (*d* = 0.79) and 4D:5D (*d* = 0.47). There are likely to be inter-correlations between these asymmetry effect sizes, for example 2D is present in two of them, as is 4D and 5D is present in three. The two largest effect sizes were found in ratios that may be independent of each other in the sense that they do not share digits (i.e. 2D:4D and 3D:5D). Summing the unsigned asymmetries of 2D:4D and 3D:5D gave a composite asymmetry with a large effect size (patient > control) of *d* = 1.04. Removing the effect of sex in a two-factor ANOVA had little effect on the magnitude of the effect size which remained large (ω^2^ = 0.231). We suggest that the unsigned composite asymmetry of 2D:4D and 3D:5D may have utility in identifying individuals that are of high risk for hospitalization resulting from COVID-19. Therefore, we have referred to it as a „Clinical Composite Asymmetry”. The utility of Clinical Composite Asymmetry as a classifier was characterized by AUC = 0.787 (good classifier). In addition, the optimum cut off point ≤ 0.087 was determined, for which sensitivity and specificity were 71% and 75% respectively with OR over 3.5. Regression analysis showed that the index > 0.087 may be a prognostic factor for hospital care for patients with Covid-19. However, to verify the prognostic value of the suggested index further studies based on larger populations in different ethnic groups are needed.

Much of the work concerning effects of prenatal sex steroids on digit ratio has concentrated on 2D:4D. However, effect sizes for 2D:4D are likely to be linked to other ratios that share 2D or 4D (i.e. 2D:3D; 2D:5D; 3D:4D; 4D:5D). The 3D:5D ratio has also been described as sexually dimorphic (males < females) and may show effects that are independent of 2D:4D^24–26^. Importantly, 3D:5D is not stable during development across age ranges from 2 to 18 years. Rather it shows a reduction with age which suggests that it may be influenced by postnatal production of androgens^24^. Comparisons between digit ratios of hospitalized patients versus controls gave small to medium effect sizes for 2D:5D, 3D:5D and 4D:5D. In so far as these digit ratios are influenced by sex steroids, this may be evidence for a link between severity of COVID-19 and prenatal (2D:4D) and postnatal (3D:5D) testosterone and oestrogen. Studies in humans and with an animal model (Golden Hamsters) have reported that SARS-CoV2 upregulates the enzyme CYP19A1 (oestrogen synthetase) leading to a profound reduction in testosterone and an increase in oestrogen in the lungs and other organs. Dysregulated sex hormones and interferon gamma (IFN-γ) levels are associated with critical illness in Covid-19 patients. In this regard, both male and female Covid-19 patients, present elevated oestradiol levels which positively correlates with IFN-γ levels (for humans^33^, for an animal model^34^). Manning and Fink^9^ reported that national values of male 2D:4D are positively related to national COVID-19 CFR’s. This led them to suggest that nations with high COVID-19 mortality have male populations that have experienced low prenatal testosterone relative to oestrogen.

However, it is more likely that the differences between patients and controls have arisen as the result of elevated levels of developmental instability in the former compared to the latter. Manning^24^ has considered the stability of all six digit ratios during growth between the ages of 2 years and 18 years. Right 2D:4D was stable but left 2D:4D was not. All ratios that included 5D showed growth-linked instability for both the right and left hands. We suggest that ratios that include 5D are „hotspots” for developmental instability that may be triggered by stressors that include rapid growth^22,24^. Recently a syndemic approach, which includes biological and social interactions for prognosis, treatment, and health policy, has been proposed. Interaction between infection with SARS-CoV-2 and an array of non-communicable diseases strongly associated with poverty, including obesity, hypertension, diabetes, cardiovascular and chronic respiratory diseases, and cancer is now considered. Moreover, syndemics are characterised by biological and social interactions between conditions and states, which increase one’s susceptibility to poor health outcomes^35^. In this respect, considering morphological signs of exposure to prenatal sex steroids and developmental instabilities (interaction between rapid early growth and stressors such as poor maternal and childhood nutrition) in patients with severe or fatal course of COVID-19 may give insight into the syndemic nature of Covid-19.

In contrast to right and left digit ratios, differences in the magnitude of digit ratio asymmetries between the right and left hand gave medium to large effect sizes. This was not apparent in directional asymmetries (R–L), perhaps because they comprise subtle deviations from perfect symmetry. Such asymmetries have been described as weakly sexually dimorphic for right-left 2D:4D (or Dr-l: with male Dr-l < female Dr-l^36^). Removing the signs from directional asymmetry (|R–L|) gave us variables that showed medium to high effect sizes in comparisons between patients and controls. Digit ratio (|R–L|) is a measure which is equivalent to asymmetry differences in digit length^22^. However, in this case we are dealing with R–L differences in morphological patterns involving two digits rather than differences between single digits of the right and left hands. It is not known whether |R–L| is sexually dimorphic across the six digit ratios. There is evidence that the phenotype of Dr-l is influenced by variation in the gene for the enzyme CYP19A1. Thus, Zhanbing et al. (2019) have reported a CYP19A1 single-nucleotide polymorphism (rs4775936) is related to variation in Dr-l in a Chinese sample^36^. CYP19A1 is important in the conversion of testosterone to oestrogen and SNP rs4775936 has been linked to the incidence of breast cancer. It may not be coincidental that up-regulation of CYP19A1 occurs in the lungs and other organs of COVID-19 patients leading to dysregulation of sex hormones (acute reduction in testosterone and an increase in oestrogen) and a marked increase in interferon gamma (IFN-γ) levels. Both are associated with critical illness in Covid-19 patients^33,34^. Further work is indicated to investigate patterns of age in digit ratio asymmetry (|R–L|). However, if it takes the form of single trait asymmetry, such as that of digit length asymmetry, it may show high levels in children which reduce with increasing age^22^. Associated with these age changes in digit asymmetry we find age dependent instability of all digit ratios that include 5D^24^. Such a pattern would suggest unsigned asymmetries of digit ratios involving 5D are sensitive correlates of developmental instability which may be negatively associated with immune system function. Such an interpretation is consistent with the view that increased asymmetry is the result of a combination of deleterious genetic and environmental factors and is defined as small, random deviations from perfect bilateral symmetry regarded as a measure of the developmental stability of the individual and phenotypic and genetic quality^23^.

We acknowledge our study has limitations: (i) Our sample size of 54 hospitalized patients is small. Obtaining good quality photographs of patients’ hands during hospitalization was sometimes challenging. A larger number would have been possible if the patient’s hands had been photocopied or scanned at discharge. However, with this latter methodology one risks missing severe cases of COVID-19 that are never discharged. We plan to extend our study, adding numbers of hospitalized patients and remeasuring digit lengths at discharge. With an increase in sample size we will consider relationships between clinical variables and digit ratios and their asymmetries. It is to be noted that within Table 4 we control for sex and report effect sizes for patients versus controls. With regard to the latter differences the effect sizes are medium to strong but none of the former are significant. This may be because sex differences in digit ratios have small to medium effect sizes. If we are correct in this we would expect that larger samples to show significant effects for sex in addition to differences between patients and controls. (ii) Additionally a further confounder may be the unknown infection status of the controls. They were recruited during the same time frame as patients hospitalized due to Covid-19 among other out-patient patients age-matched and based on their negative history of any symptoms of Covid-19 infection. Such appointment of controls may have resulted in a heterogeneity of this group such that they may have included non-infected individuals as well as non-symptomatic but infected or past-infected individuals. However, this does not invalidate the idea of this study which was focused on “markers” of symptomatology related to hospitalization. It would be beneficial to perform such analysis comparing symptomatic versus asymptomatic (or mildly symptomatic treated out-patients) but infected patients. However, this was not possible during the first waves of Covid-19 as there was no nation-wide testing in Poland (in general only symptomatic individuals were tested). In this regard our results may be biased by behavioral factors—i.e. the way participants prevented infection. (iii). We were not able to make comparisons between individuals who had been vaccinated and those who have not. This was because our data were obtained during the first wave of Covid-19, so none of the participants (patients and controls) had been vaccinated. In this regard, it would be of great interest to compare the efficacy of the vaccine in individuals with high and low values of the „Clinical Composite Asymmetry”. The prediction would be that vaccine efficacy would be low when „Clinical Composite Asymmetry” is high and high when „Clinical Composite Asymmetry” is low.

In conclusion, we have found differences in digit variables between patients hospitalized for COVID-19 and controls. Overall, our findings point to high levels of developmental instability in the former compared to the latter. Our focus was on six digit ratios and for each we considered right and left ratios and their asymmetries (signed and unsigned). We found differences in digit ratios between patients and controls that were focused on ratios that included 5D. The effect sizes were small to medium. Unsigned asymmetries of four digit ratios, including three that involved 5D, yielded medium to large effect sizes with patients > controls. The largest of these asymmetries were for 2D:4D and 3D:5D. A „Clinical Composite Asymmetry” for these two variables gave an effect size which may have some utility in identifying individuals who have experienced high developmental instability. Thus, this „Clinical Composite Asymmetry” may enable us to identify individuals who are likely to experience severe COVID-19 and those who may not.
